# Integrated liaison psychiatry services in England: a qualitative study of the views of liaison practitioners and acute hospital staffs from four distinctly different kinds of liaison service

**DOI:** 10.1186/s12913-019-4356-y

**Published:** 2019-07-25

**Authors:** Keeble Jasmin, Andrew Walker, Elspeth Guthrie, Peter Trigwell, Alan Quirk, Jenny Hewison, Carolyn Czoski Murray, Allan House

**Affiliations:** 1 Department of Digital, Media, Culture and Sport, London, UK; 2 Clinical Research Network National Coordinating Centre, National Institute of Health Research Clinical Research Network, Leeds, UK; 3 Leeds Institute of Health Sciences, University of Leeds, Leeds, UK; 4 National Inpatient Centre for Psychological Medicine, Leeds and York Partnership NHS Foundation Trust, Leeds, UK; 5 College Centre for Quality Improvement, Royal College of Psychiatrists, London, UK

**Keywords:** Consultation-liaison, Liaison mental health, Liaison psychiatry, Hospital psychiatry, Qualitative, Health services research

## Abstract

**Background:**

Liaison psychiatry services provide mental health care for patients in physical healthcare (usually acute hospital) settings including emergency departments. Liaison work involves close collaboration with acute hospital staff so that high quality care can be provided. Services however are patchy, relatively underfunded, heterogeneous and poorly integrated into acute hospital care pathways.

**Methods:**

We carried out in-depth semi-structured interviews with 73 liaison psychiatry and acute hospital staff from 11 different acute hospitals in England. The 11 hospitals were purposively sample to represent hospitals in which four different types of liaison services operated. Staff were identified to ensure diversity according to professional background, sub-specialism within the team, and whether they had a clinical or managerial focus. All interviews were audio-recorded and transcribed. The data were analysed using a best-fit framework analysis.

**Results:**

Several key themes emerged in relation to facilitators and barriers to the effective delivery of integrated services. There were problems with continuity of care across the secondary-primary interface; a lack of mental health resources in primary care to support discharge; a lack of shared information systems; a disproportionate length of time spent recording information as opposed to face to face patient contact; and a lack of a shared vision of care. Relatively few facilitators were identified although interviewees reported a focus on patient care. Similar problems were identified across different liaison service types.

**Conclusions:**

The problems that we have identified need to be addressed by both liaison and acute hospital teams, managers and funders, if high quality integrated physical and mental health care is to be provided in the acute hospital setting.

**Electronic supplementary material:**

The online version of this article (10.1186/s12913-019-4356-y) contains supplementary material, which is available to authorized users.

Liaison psychiatry is the sub-specialty of psychiatry that focuses upon the interface between psychiatry and non-psychiatric clinical services [1]. Most commonly this involves psychiatric provision to the acute general hospital but can include other specialist hospitals and also primary care [2]. Several different models of liaison psychiatry exist with differing degrees of penetration into the general hospital and different styles of working [3]. The most common types of service in the UK are hospital based teams that provide on demand consultation and treatment for patients in acute hospital settings, with some also providing out-patient work or specialist in-reach to specific medical teams/specialties. The term psychosomatic medicine has been used to refer to liaison services, although this term is now used more specifically to refer to services that provide treatment for patients who have physical and mental co-morbidities. Liaison services provide treatment for all patients in the acute hospital with mental health problems including those with physical and mental co-morbidities, self-harm, dementia, alcohol and drug related problems, behavioural disturbance etc., There is growing, although still somewhat limited, evidence that liaison psychiatry services are effective [4] and may lead to cost reductions in healthcare [5].

In the United Kingdom, liaison psychiatry is a sub-specialty of general psychiatry. Psychiatric higher trainees can gain an endorsement in liaison psychiatry by undertaking a 12 month training, but this is not mandatory. There are no specific training requirements in liaison psychiatry for physicians or nurses. General practice trainees are encouraged to spend six months in a psychiatry training post which can include liaison psychiatry,

A recent survey of all acute general hospitals in England reported that 168 out of the 179 acute hospitals with an emergency department had some form of liaison psychiatry service [3]. Further expansion is planned, with a national target that at least 70% of all acute hospitals in England will have liaison services staffed to key commissioning standards by 2023/2024 [4]. This will most probably mean an increase in staffing for many services, and there is an expectation that the cost of further development will be offset by financial savings, predominantly via a reduction in either length of stay or re-admission rates.

Most liaison services in England are commissioned, managed and delivered as part of mental health services rather than general hospital services. The core of liaison work, however, involves close collaboration with general hospital staff to ensure the best care is provided for people with mental and physical health problems. The recent National Confidential Enquiry into Patient Outcome and Death (NCEPOD) into standards of care for people with mental and physical health problems in the general hospital highlighted various problems with current provision and quality of care [5]. One of its key recommendations was that liaison staff should be part of the general hospital multidisciplinary team - fully integrated into the general hospital system.

Integrated health systems are considered to provide superior performance in terms of quality and safety as a result of effective communication, and shared decision-making, although these outcomes have not been fully demonstrated [6, 7]. Integrated care has been defined as, “a coherent set of methods and models on the funding, administrative, organisational, service delivery and clinical levels designed to create connectivity, alignment and collaboration within and between the cure and care sectors” [8]. There is no accepted conceptual model for health systems integration but key principles affecting clinical delivery include: patient focus (ensuring the patient receives the right care in the right place at the right time); inter-professional team working; information systems which enhance communication capacity and information flow across integrated pathways; and an organizational culture that is congruent with a shared vision of care [9].

To explore the degree to which current liaison psychiatry provision in England is indeed integrated into physical health care, we undertook the present study interviewing liaison mental health staff working in general hospital liaison services and acute hospital staff who had experience of referring to liaison services. In this paper, we focus on facilitators and barriers to providing high quality, multidisciplinary care in relation to each of the above areas, and present the themes that arose from this work according to the key principles of integrated care outlined by Suter and colleagues [10]: co-ordinated transitions in care across the continuum of care; patient focus; presence or absence of primary care structures to support discharge; inter-professional team working; communications systems; organizational goals and objectives aligned across sectors; physician integration within teams; and attainment of goals and objectives are supported by funding and human resource allocation.

## Methods

This work formed part of the first phase of a programme funded through the NIHR Health Services and Delivery Research scheme to evaluate the cost-effectiveness and efficiency of different configurations of liaison psychiatry services in England (LP-MAESTRO) (http://www.nets.nihr.ac.uk/projects/hsdr/135808/#/). Ethics approval was received from the North of Scotland Ethics Research Service (REC reference: 15/NS/0025) and NHS Trust level approvals obtained. All participants provided written informed consent. We have followed the Consolidating Criteria for Reporting Qualitative Studies (COREQ guidelines) [11].

### Research team and reflexivity

The interviews were carried out by two researchers AW (male) and JK (female); one from the LP-MAESTRO project itself (AW) and one from the College Centre for Quality Improvement (JK), the Royal College of Psychiatrists, both qualified by experience and training. Neither were involved in the delivery of clinical services and were not mental health professionals.

### Design

A cross sectional qualitative study of liaison mental health staff and acute hospital staff about their experiences of liaison psychiatry services.

### Theoretical framework

The data were analysed using a best-fit framework analysis [12]. The key themes that emerged were grouped, where relevant, according to Suter and colleagues [9]. The topic guide did not make reference to the Suter principles, which were used after the initial framework analysis to structure the themes.

### Participant selection

A sample of liaison services was identified using the following procedures. All 168 hospitals in England that had a liaison service were asked to complete an on-line survey about liaison psychiatry services [3]. A member of staff from each of the 168 services (100%) completed the survey. The survey data were analysed using a latent class model to perform clustering of the hospital services, which is described in detail in a separate publication [13]. Four clusters were identified, none of which mapped onto current liaison classifications such as CORE [14] (types of liaison services defined by commissioning guidelines from NHS England) or Rapid Assessment Interface Discharge (RAID) teams [15]. The four clusters basically comprised:

- small services, which in the main did not provide 24/7 cover or out-patient services (Cluster 1) (*n* = 46)
- services that provided 24/7 acute cover but very little non-acute work (Cluster 2)(*n* = 35)
- services that covered the acute work but offered non-acute care and ran outpatient clinics (Cluster 3) (*n* = 43)
- services that were not exclusively focused on the acute care pathway, provided non-acute care and had separate adults of working age and older adult teams (Cluster 4) (*n* = 44)

All 168 liaison services were then contacted by email to determine their interest in participating in the present qualitative study. Eighty-six services (51%) did not reply, 6 indicated initial interest but then provided no further contact, 33 declined (18%), and 17 services (10%) were excluded as they had undergone significant change since the initial survey.

This left 30 services (18%), staff from which were invited to one of two workshops at which the aims of the present study were explained, in addition to those of the rest of the programme. Representatives from 13 services (8%) attended the workshops and 11 of these services were purposively sampled, informed by the cluster analysis, to ensure that each of the 4 clusters was represented by between 2 and 4 liaison services.

In each of the 11 services, we sampled staff identified to ensure they were knowledgeable about the realities of service provision and diverse in experience according to the following characteristics: professional background, sub-specialism within the team, and clinical or managerial focus. The local Liaison Psychiatry [16] lead, received a list of potential interviewee roles from the project manager (AW). The LP lead then contacted the LP team and asked if anyone did not want to participate. The LP lead then provided contact details by emailing potential interviewees with an introductory email and information sheet and participant information sheet. The LP lead scheduled interviews and informed consent was obtained in writing at the time of the face to face interview.

We also interviewed acute hospital staff served by the liaison services about their experience of the liaison team. We sampled staff from regular referrers to the liaison service, obtaining as wide a range of different referrers as possible.

### Data collection

Seventy three participants were interviewed in-depth, individually, using a semi-structured topic guide informed by similar approaches in for example realist evaluation focusing on events or processes rather than norms, values or presumptive theories [17]. With permission, all interviews were audio-recorded. The topic guide was developed specifically for this study and consisted of a list of key topic areas with open ended questions and additional prompts. The topic guide is available an additional file (Additional file 1). Exact wording was left to the researchers to tailor to the individual participant. The following areas were covered:

- Introductory questions identifying the role of the participant and their professional history.
- Participant’s understanding of the structure of the liaison psychiatry service, and account of where they worked within it.
- Participant’s account of types of clinical work undertaken, and outcomes aimed for.
- Nature of working relationships in the acute hospital, in the associated Mental Health Trust, and with other professionals and agencies with whom the participant interacted.
- Participant’s understanding of the origins of the service, current influences on its form and functions.
- Participant’s views on desirable changes and ways to achieve them.

The interviews took place between October 2016 and January 2017 at the participant’s place of work and were recorded and transcribed verbatim. The interviews lasted between 30 min to 90 min.

### Data analysis

The data were managed using the qualitative software package NVivo (version 11). Transcripts were systematically summarised. JK and AW have a working knowledge of mental health but are not psychiatrists and did not have prior contact or involvement with liaison psychiatry services. The following researchers were involved in the qualitative analysis: JK, AQ, AW and AH. There is a potential bias in that one of the 73 participants interviewed (EG) was a member of the research team, however, EG did not have access to the interviews or transcripts and was not involved in any of the qualitative analyses, or any discussions about the analyses. These analyses were written up in the form of a report by the qualitative team, with the lead author JK. EG was not involved in this work. There were no repeat interviews. Transcripts were not returned to participants for comment or correction.

### Reporting

Quotations from participants are used in the results section to illustrate themes.

## Results

### Sample characteristics

Seventy three in-depth qualitative interviews were conducted with professionals from the liaison services (*n* = 60) and acute trust colleagues (*n* = 13), who had experience of working with the liaison teams. Services were of varied size and had diverse configurations and staffing levels, as indicated by membership of the 4 different clusters. The number of participants interviewed therefore varied between services -between 4 and 11 interviews were conducted at case study sites.

There were 2 hospitals in Cluster 1 (8 liaison mental health staff), 2 hospitals in cluster 2 (10 liaison mental health staff and 1 acute hospital staff member), 3 hospitals in cluster 3 (15 liaison mental health staff and 5 acute hospital staff) and 4 hospitals in cluster 4 (26 liaison mental health staff and 8 acute hospital staff).

A greater proportion of liaison staff were interviewed than acute hospital staff as our main focus was on the liaison teams. Fewer staff were interviewed in Cluster 1 hospitals than the other hospitals as the teams were much smaller, and despite efforts, we were unable to interview an acute hospital staff member in these two hospitals.

#### Findings

Table 1 shows the original nascent themes and how they were grouped according to the ‘nature of the liaison teams’ and the key principles outlined by Suter and colleagues [16].

**Table 1:** Original themes that emerged from the data, grouped according to the ‘nature of the liaison teams’ and the Principles of Integrated Care by Suter and colleagues [14]

Original themes	Groupings
Role of liaison psychiatry Service development and history Diversity of work Components of liaison services organised in different ways Aims and purposes of services Types of patient problems seen by liaison services	The nature of the liaison teams
	Principles of Integrated Care (Suter et al., 2010) [10]
Confusion about the referral process to liaison services Inappropriate referrals including patients with no mental health problems Onward referral and signposting	Co-ordinated transitions in care across the continuum
Value of assessments Response to non-engagement Helping patients to manage mental health issues better via informal learning opportunities Description of the clinical process Continuing support and assessment of needs Brief interventions or follow-ups during admission Medication prescribing Preventing suicides Avoiding exacerbating long-term mental health problems Improving quality of life Improving adherence to medication for physical health problems Improving clinical outcomes for patients	Patient focused care
Gaps in service provision Interaction with external psychiatry teams Difficulty in accessing community services	Primary care network structures in place
Relationships with referrers Collaborative working Training and education- Influencing team members and acute service colleagues	Inter-professional team working and team effectiveness
Notes and recording systems Time taken to record information Duplication of record keeping Absence of shared record keeping	Information systems which enhance communication capacity and information flow across integrated pathways
Physical space and identity Problems with commissioning of liaison teams Visibility	Organisational goals and objectives aligned across sectors
Hierarchies in mental health and acute trusts Alternative perspectives regarding training of acute staff	Physician integration within care teams and across sectors
Reduction in length of stay Limiting time patients spent in ED Better communication with commissioners Promoting safe and efficient services-mismatch between staffing levels and volume of referrals Specialism versus generalism Influencing commissioners to expand systems Influencing mental health trust managers National models and structures Perceived prioritisation by commissioners Promoting safe and efficient services	Attainment of goals and objectives are supported by funding and human resource allocation
What liaison services wanted to change- internal delivery processes and structures Challenges in delivering training Address mental health stigma amongst acute services colleagues Alternate persectives about training	Do not map onto a specific principle

### The nature of the liaison teams

As is common nationally, the liaison psychiatry services in our sample were provided by Mental Health Trusts but served Acute Hospital Trusts. The liaison psychiatry services were teams of psychiatrists and liaison practitioners - most commonly mental health nurses, sometimes with one or more psychologist, social worker, therapist, or physician associate. They took referrals from physical health colleagues in acute hospitals - Emergency Departments [13], acute medical units and general wards, out-patient settings and primary care. There was a broad agreement amongst the liaison team members interviewed of the aims and purpose of the services. These were to help with the management of patients with co-morbid physical and mental health problems, and where relevant, facilitate the safe discharge of patients to appropriate settings.

Seven main types of clinical scenarios emerged involving people with: self-harm; delirium and dementia, typically in people with physical health problems; severe mental illness co-existing with physical health problems admitted to hospital or seen in the ED; mental health problems arising as a consequence of long-term physical illness or its medical treatment; physical illness exacerbated or caused by mental health problems for example through poor adherence to treatment regimes, or by other mechanisms; unexplained persistent physical health symptoms, the severity and chronicity of which were disproportionate to suspected underlying disease mechanisms; physical or psychological consequences of alcohol or drug misuse.

Our participants recognised social influences on the referral patterns - vulnerable groups including homeless people and those who experienced domestic abuse were particularly likely to be referred, and hospitals which served particularly large ageing or student populations experienced more referrals from these groups.

Several themes emerged in relation to facilitators and barriers to the effective delivery of services. These themes were considered, and discussed within the research group, and are presented according to key principles of integrated care outlined by Suter and colleagues (2017) [16], as nearly all were captured by this framework. Themes that fell outside of these principles included those involving internal processes within the teams for the assessment and treatment of patients, which will be covered in a separate publication.

### Coordinated transitions in care across the continuum

Problems with coordinated transitions in care emerged in relation to referrals to the liaison teams from the acute hospital teams, and from the liaison teams to community/primary care services.

i) Problems with referrals to the liaison services

Some acute trust professionals described frustration with a perceived lack of responsiveness from liaison teams. This was more common in the larger liaison services (20+ team members) that were likely to have separate components leading to confusion and frustration when referring a patient. There was also confusion about the location of responsibility between hospital-based liaison teams and community mental health services. As a participant from an acute trust explained:

*‘I have once lost my rag on the phone and said, 'I don't bloody care. Somebody from mental health needs to come and see this patient. You can argue between yourselves whether it's the inpatient team or the community team. That's not my job.’* (sF_p01)

Conversely, some liaison psychiatry professionals described frustration when they received referrals from acute service colleagues for patients who they thought were inappropriately referred - for example, a patient who had received some distressing news or had had a recent bereavement, and may have been visibly distressed but not suffering from a mental illness. Liaison staff speculated that acute hospital colleagues referred patients with these types of problem because they lacked confidence. Liaison psychiatry professionals also felt that it might not be sustainable to accept referrals for what they described as more straightforward cases which they felt acute hospital teams should be able to manage themselves, for example, patients with delirium. As a liaison participant reported, with reference to managing delirium on wards:

*‘If we always do everything then they don’t learn to do it for themselves! …sometimes we’re inundated with referrals so if we see everything… well actually sometimes you can’t so sometimes they get left waiting a little bit’* [sI_p06].

While liaison staff felt it was within their remit to help acute hospital colleagues rule out mental health/psychiatric problems, they thought acute hospital colleagues should be able to manage patients who were emotionally distressed but not mentally ill, and those with relatively straightforward mental health problems.

Liaison staff who worked in services representative of all the different clusters described a higher rate of inappropriate referrals from new junior doctors who were particularly risk averse.

ii) Difficulty in accessing community services

Several barriers emerged in being able to access community services. Liaison staff expressed concern that patients experienced difficulties and delays in accessing external services following liaison referral. One explanation was perceived inefficiencies in hospital administration systems such as delays in sending out referral letters.

Staff, however, also reported a perceived unreceptiveness of community services to patients who were referred. When referrals were managed by gatekeepers, there were concerns that patients might subsequently be told they did not meet the threshold for a particular service, and no clear arrangements or plan could be established prior to discharge. Referral to some services involved long waits, resulting in what staff members felt was an unacceptable, circular process.

For example, a liaison participant described the case of a young person who had been referred to the local Improving Access to Psychological Treatment Services (IAPT) following self-harm. The IAPT service, however, felt she was too high risk for them to manage and suggested placing her under the care of the Community Mental Health Team (CMHT). Whilst this referral was being expedited the young person’s state of mind deteriorated and she was re-admitted to hospital following a suicide attempt. The liaison team member was frustrated by this incident.

*‘I mean we feel super frustrated. Incredibly frustrated and just really sad for this girl who just needed … a little bit of talking therapy and she needed some actual practical support. She's 18 and it was all a bit horrible socially. Instead she got passed around which just fed into those feelings of rejection’* [sA_p03]

There were also examples of older people, who liaison staff suspected had dementia and were caught up in protocols between memory clinics and community mental health teams. This presented difficulties for liaison psychiatry services when these patients were repeatedly admitted to acute hospital beds, but had not been given a diagnosis of dementia. Staff reported it was inappropriate to diagnose dementia when a patient was acutely unwell, so liaison staff felt frustrated that they were unable to develop appropriate care plans.

### Patient focused care

Liaison staff valued highly the role of detailed psychosocial assessments as part of their work, and assessments were viewed as an important step to ensuring patients received the best possible care. Assessments were described as typically lasting up to an hour and involved exploring reasons for admission, the person’s view of their own circumstances and risk, resulting in a detailed picture of the patient’s current situation. Assessments were regarded as therapeutic in their own right, providing an opportunity for patients to feel heard and understood. Liaison staff reported some patients showing visible signs of improvement during the assessment interview:

*‘Often we’ll get feedback from the patients saying it’s really nice to have somebody to talk to. It’s really great that you really listened to me today. Because we kind of expect that we’re going to spend 40, 45 minutes with somebody, the fact that we sit down and they get a chance to talk. They don’t get that on a really busy ward. Em… you know the doctors and nurses will come in, get the information they need quite quickly and go again. So we often get told that you’re the first person that’s listened to me.’* [sI_p06]

Liaison staff felt that assessments should be of a high quality and carried out by appropriately trained staff, who had the right *‘tone, volume and attitude’* to facilitate engagement and, encourage the patient to speak freely. Participants reflected that patients sometimes found it easier to talk to nurses, or other non-medically trained professionals, because they perceived them as being lower down a hierarchy of doctor – nurse – patient.

Some staff provided continuing treatment for patients admitted to acute hospitals over longer periods. For example, at one service this involved mental health nurses regularly visiting older adult patients or those on stroke wards to provide encouragement with eating and rehabilitation; two vital components for ensuring recovery. Teams that had psychologists, therapists or mental health nurses trained in specific interventions (like cognitive behavioural therapy) offered brief interventions while the patient was in an acute hospital bed, or a follow-up appointment after they had been discharged.

Liaison staff believed a large part of their overall work was to teach patients how to manage their mental health problems better. Liaison services described this as providing basic psychological education. This was true of all settings, but particularly the focus of out-patient clinics. One service provided home-visits for COPD patients, primarily focused on education and management. This involved a minimum of two to three sessions, which was used to teach patients breathing and relaxation techniques.

### Primary care network structures in place

Liaison staff described gaps in service provision after discharge. This was particularly evident for patients with medically unexplained symptoms (MUS). Several staff expressed concern about no suitable services for this group of patients, who had persistent and disabling symptoms, and who required more intensive treatment than the brief, shorter term packages available. As a participant explained:

*‘They need long-term psychotherapy with a reliable, skilled practitioner, probably Band 8A (highly skilled) , who will see them every week and support them in making small, small changes. What they get is a Band 5 (moderately skilled), if you're lucky, IAPT person … and it's not therapeutic for anybody concerned.’* [sB_p03]

Participants reflected that there simply was not the funding available for this sort of treatment, which relied on incremental change over a period of months. Some staff reported making direct individual appeals to their Clinical Commissioning Groups to provide appropriate treatment for certain people with severe problems. A lack of appropriate treatment facilities was a source of frustration for staff, who worried that these patients would remain seriously unwell and could end up having unnecessary investigations or treatments, which would be detrimental to their overall health.

Whilst staff who worked in liaison services with dedicated out-patient clinics could provide some provision for these patients, they worried about their ability to provide comprehensive care. Staff who worked in liaison out-patient clinics did see patients with severe MUS:

*They [CMHTs] would not know how to treat patients with complex medically unexplained symptoms on high-dose opiates or they wouldn't know how to treat patients with non-epileptic seizure disorder. So, I think we see a different group of patients, so we provide the therapy because the CMHTs wouldn't offer these patients treatment* [sF_p04]

A suggestion from some liaison staff was that experienced liaison clinicians should be embedded either into community mental health teams, or community/primary care liaison services and should be resourced to provide long-term treatment. While liaison staff acknowledged this was costly, one view was that non-intervention was even more expensive, if subsequent contacts with acute and mental health services, were taken into account.

### Inter-professional team working and team effectiveness

Generally, staff described good working relationships with colleagues from different specialties. Close working relationships were described between liaison psychiatry services and acute hospital colleagues, especially those that had developed over years. This sort of relationship was underpinned by the liaison service having credibility through a detailed understanding of both medical and mental health. As an acute trust participant described their relationship to their liaison service:

*‘[Previous liaison psychiatrist] had a good team around him and provided good rapport, can relate to the problems on the shop floor. [Current liaison psychiatrist], who came up through the system…you know, there's a good knowledge of the individual and therefore there's also a good working relationship.’* [sJ_p05]

Liaison services and acute referrers who had worked in the same acute hospital over decades felt that this length of time facilitated productive relationships and led to fewer barriers to making referrals; they understood who and what the liaison team did, and how to contact them.

Liaison psychiatrists felt it was part of their role to work collaboratively with acute hospital consultants to implement agreed plans in acute hospitals. For example, at one service (based in an acute hospital), a liaison psychiatrist explained how they would find the consultant in charge of the patient’s management plan and discuss the patient’s management in detail.

In general, it was felt that those who worked in liaison psychiatry services, regardless of background, needed to project professional competence. A consultant psychiatrist reflected:

*‘If you go on the wards, you've got to get the notes, read the notes, discuss the person with the nursing staff and the medical staff, and often they'll be just talking about people with a consultant physician or consultant surgeon so you have to have some confidence in what you're saying’* [sH_p03]

Training and education was also seen as a part of building collaborative working.

*‘A lot of my job is about training and understanding, and trying to have a presence there, and trying to get people to think about things’*. [sF_p06]

Close working was also described in relation to prescribing medication. Advice and guidance about the use of psychotropic medication in physically unwell patients was provided by liaison services, principally by liaison psychiatrists. In many acute hospitals, liaison psychiatrists were not able to prescribe medication, as they were employed by a separate organisation (e.g. a mental health trust), but provided a key role in providing appropriate advice to medical and surgical colleagues.

Acute staff referred to an informal osmosis of skills in mental health communication if inter-professional relationships were productive.

*‘I've learned a lot from it actually. Little things like the vocabulary you use, the ways you describe some of the symptoms that the patient is complaining of, and describe what's causing it…So you might say that we know that the symptoms you have are very real, we know that you're experiencing these symptoms and the challenge we're facing is the way that your brain interprets those symptoms. One of the examples they used, which I've stolen and used again, is, say, when you stub your toe and you're in a bad mood it feels a lot worse than when you stub your toe and you're skipping on the beach. So it's just a little explanation as to the way the mind and pain work together, so it was a really helpful explanation which I have stolen’.* [sK_p06 ]

### Information systems which enhance communication capacity and information flow across integrated pathways

i. Time taken to record clinical contact

Liaison team members expressed concerns about the amount of time required to adequately document and record clinical contact with patients. This could take up to two hours in some instances and limited the responsiveness of the team to other referrals.

ii. Absence of a shared patient record system

Both acute hospital staff and liaison team members described frustration with the absence of a shared patient record system.

Liaison staff described having to enter similar/duplicate data on mental health systems and acute hospital systems. If acute trusts still used paper clinical records, this would also involve writing in the clinical notes. This duplication added to an already lengthy process of clinical documentation.

Acute Trust staff described being unable to access mental health records for their patients, which interfered with providing good quality care. For example, a geriatrician who wanted to know if there was a pre-existing dementia diagnosis felt this information should be freely available to the acute trust and did not perceive it as sensitive information. Other examples involved the management of patients with multiple presentations to acute services with medically unexplained symptoms, where sharing of mental health information would have facilitated improved care.

Some services developed ways of circumnavigating information barriers, for example, foundation doctors (medical trainees in their earliest years of training), who were attached to liaison services for brief training periods, could access both mental health and acute trust systems. At larger liaison services, where team members were more likely to attend multi-disciplinary team meetings in the hospital, it was easier for team members to check mental health records for acute services colleagues, and report back at the next meeting, although this was time consuming and cumbersome.

### Organizational goals and objectives aligned across sectors

i) Lack of visibility

There was little evidence that acute hospitals and liaison teams had a shared vision of integrated care. This was most stark in hospitals where the liaison team received no referrals from certain parts of the acute service. Liaison staff reported a lack of visibility, for example, not receiving any referrals from surgical wards. While liaison teams in this situation reported making efforts to promote their services more widely, they also feared this may lead to levels of service demand that they were insufficiently resourced to manage.

ii) Commissioning of liaison teams to only cover certain parts of the acute hospital

Another example of a lack of a shared vision, was commissioning of liaison services to only certain parts of the acute hospital. This was more common in the smaller liaison teams and was sometimes due to historical reasons rather than a result of planned commissioning. However, in some services funding had been cut, effectively de-commissioning referrals from some departments in the hospital. This left staff in a difficult position, as a participant explained in relation to referrals from maternity services for which the team were not commissioned to provide a service:

*‘I would never turn down a maternity referral because of the potential risk involved…I mean you've got a mother and a child, and there have been a lot of high profile and very tragic cases of late where there's been a puerperal psychosis involved. The extra stresses on the family with a new baby, the possible medical problems as well, I think it just makes it out as very high-risk.’* [sB_p01]

Staff also worried, however, about the repercussions of doing this work, if, for example, taking a non-commissioned referral meant they were delayed in responding to a patient from a commissioned department. Breaching the four-hour response time in emergency departments was a concern because it was a key metric of success used by commissioners for some teams. This caused feelings of frustration for those in those teams, as a participant reported, ‘w*e feel so frustrated most of the time because we can’t do what we are trained for’.’*[sA_p02].

There were varied views on how liaison psychiatry services should respond to this dilemma. One perspective was that liaison professionals should prioritise safety (of patients and staff), regardless of formal commissioning arrangements, and take referrals they perceived as higher risk. Clinicians linked this to their ethical obligation to help anyone in need. An alternative position was that liaison services should limit themselves to what they were commissioned to do, which was felt to avoid unnecessary job strain. These dilemmas were not a problem for teams that were commissioned to cover the whole hospital.

iii) Lack of physical space

Several different teams reported not having enough office space for supervision, meetings, and administrative work, as well as limited access to suitable rooms for assessing patients. This was practically and clinically challenging for liaison staff. For example, a participant described having to conduct assessments in a room adjacent to a children’s ward, with poor sound proofing, so children could hear loud or agitated patients in the room. This also made it difficult to offer patients privacy.

There was a link between the physical space made available to liaison teams and their sense of identity. When liaison teams felt they lacked appropriate physical space to perform their role they sometimes felt excluded and dispirited. In contrast, when liaison teams felt accommodated by acute trust colleagues they reported feelings of integration and cohesion with acute services colleagues.

For example, a liaison service had a dedicated team for the assessment of people with self-harm in the emergency department. This team shared an office with the emergency department staff. There was a strong suggestion that this team felt fully integrated with their acute trust colleagues.

### Physician integration within care teams and across sectors

Different hierarchies operated in Acute and Mental Health Trusts. Liaison psychiatry staff typically felt that Acute Trusts were generally more hierarchical than their own Mental Health Trusts.

*‘There is a hierarchy that exists in health and basically the consultants over there sometimes perhaps don't respond as well to the nurses over there as they would respond to a consultant psychiatrist so having a consultant psychiatrist in your team, when certain things are going on and communication at a certain level is actually to an advantage of the team I think, and I think that is actually to do with the hierarchy that exists within health…Yes, I think that it exists less in mental health but having worked in A & E for a long time so a hierarchy exists … and if the consultant says something then that is what happens.’* [sH_p04]

In Acute Hospitals, particularly EDs, liaison nurse practitioners tended to assess uncomplicated new referrals and develop plans independently of consultants. Practitioners only consulted a psychiatrist if they had specific concerns. This approach relied on liaison practitioners being able to act with a high degree of autonomy. It was therefore problematic for liaison nurses when their acute hospital colleagues were more receptive to advice from psychiatrists.

Acute Trust colleagues recognised this response to liaison practitioners and agreed that it was, to some extent, linked to hierarchies. However, they also highlighted specific occasions where input from a psychiatrist was perceived as necessary but absent, for example, if they wanted advice on drug interactions. In these instances, acute referrers felt it would have saved time if a consultant liaison psychiatrist had been able to see and manage the case.

### Attainment of goals and objectives are supported by funding and human resource allocation

Many liaison team members described rapid changes in liaison services, largely influenced by national drivers and targets set out in plans to expand liaison services on a nationwide basis [4]. On the other hand, there was a sense that much of this change was driven by key clinical specialists in particular teams, who were responsible for driving local expansion through direct and frequent contact with commissioners, and aligning service development with government targets.

Liaison staff described many instances of internal dilemmas in teams created by a need to make decisions about the best use of limited resources. As an example, one dilemma was between specialism and generalism. Staff expressed differing views about the extent to which liaison practitioners should be able to specialise by age and/or setting. Those in favour of specialisation: argued that different liaison professionals are more/less suited to different types of clinical work. For example, liaison nurses who enjoy working in the emergency department were characterised as liking autonomy, positive risk-taking and working at a fast pace. Those who preferred working with older adults were characterised as liking to develop relationships with their patients over a longer period of time and working at a slower pace. Those who strongly identified with either extreme were particularly averse to working outside of their preferred clinical area, and expressed concern about their ability to carry out the new role safely.

### Differences in views from staff in different hospital clusters

There was little evidence of major differences in responses between the different types of liaison services. More concerns about specialism were expressed in larger services with separate components (e.g. older adults team), in comparison to the other service types, and there also appeared to be greater confusion about referral processes. However, there was remarkable consistency across service types concerning the problems or difficulties that teams described.

## Discussion

This study outlines some of the barriers to achieving integrated physical and mental health care by liaison mental services in the acute hospital setting. These barriers are similar across the four distinctly different types of liaison mental health services that are currently in existence in England.

In general, liaison team members and hospital staff report good personal working relationships, with a focus on patient centred care but staff report major problems with coordinated transitions in care, referral to primary/community care services, information sharing and shared organisational goals and objectives.

One of the driving purposes for the current round of investment in England is the hope that such a move will result in a reduction in length of stay, particularly for older adults with physical and mental co-morbidities [4, 17–20]. This is dependent, however, upon seamless transitions of care between the hospital and community services, and the existence of high quality community mental health services. Investment in hospital liaison services alone, without additional investment in crisis teams and services for the elderly with mental health problems, will risk patients being unable to access community services because thresholds are too high, resources are too limited, and there is a focus on gatekeeping. These factors make continuity of care challenging. Good communication with primary care is also important as many patients seen by liaison services can be managed by their general practitioner (GP). Speaking directly to the GP as opposed to written communication results in better care and patient outcomes [23].

An alternative possible way to solve this problem of bridging mental and physical healthcare is to embed mental health practitioners within physical healthcare pathways, or develop and extend liaison services to primary care [2]. Such services have been tried and may help some problems without fully resolving others – for example they raise problems of scaling up, redefining patterns of team working, and they do not resolve the questions related to information-sharing or the need to balance the demands of acute rapid-response referral work with those of slower-stream shared care in embedded teams.

The participants we interviewed did not mention the existence of any protocols to help clarify pathways and referral systems, other than those used by community psychiatric teams which were used to set high thresholds for referral to these services. Acute hospital staff voiced frustration at not knowing which service to refer to, if there was more than one psychiatric service within the same hospital (e.g. an adult of working age team and an old age team). A single point of access and clearer referral systems and protocols would help reduce some of the frustration felt by acute hospital staff.

The liaison practitioners in this study reported that a substantial amount of their time was spent in record keeping, limiting their ability to respond to the urgent requirements of acute hospital staff who were used to a more rapid throughput of patients. Mental health electronic assessment systems have been designed in the main for patients with severe mental illness, who are managed by mental health staff with relatively small caseloads, who see their patients on a regular basis, over long periods of time (months and years). Such systems are inappropriate for liaison mental health teams who operate in large volume, low contact services. It is clearly an inefficient use of staff time if they spend twice as long recording information about a patient as they do in face-to-face therapeutic contact with that person. Mental health systems now usually involve completion of complex risk assessments, even though, such assessments have little or no predictive validity, and there is no evidence that they reduce harms associated with mental illness [21–25].

Our findings suggest that mental health recording systems for liaison psychiatry are not fit for purpose and are severely affecting the responsiveness of teams. Liaison psychiatry electronic systems should be tailored to meet the requirements of high volume, low intensity work. They should not use systems that have been designed for the assessment and long term management of patients with severe mental illness in the community or in-patient setting. A reduction in the time taken to electronically record assessments is one obvious area where liaison services can become more efficient and effective as it would free up staff time to have more therapeutic time with patients, and more patients could be seen. As alterations to standardised information systems are difficult and can potentially lead to a worsening of data transferability and efficiency, any changes should be fully piloted and evaluated in a variety of centres before widespread introduction.

There is a move in the NHS towards shared electronic health records, and increased use of electronic devices to record information. These initiatives are welcome but must be accompanied by a shift away from a focus on risk assessment to safety management, with more focused data recording.

Our findings also suggest that there needs to be a way of sharing information across systems for liaison psychiatry services and acute hospitals. Whilst there are genuine and real concerns about patient confidentiality, this must be balanced with concerns about staff not having access to important information about patients, which may adversely affect their welfare. Any sharing of information will need to comply with changes to the General Data Protection Regulation which came into force in the UK in May 2018. Separate recording systems are also likely to result in mistakes in medication recording which again could have serious consequences. Liaison services which are managed by Acute Hospital Trusts may use the acute hospital’s information system, but if these services are unable to access and share information in separate mental health systems the barriers remain – just in a different place in the system.

This is a complex area, but it would be helped by the development of bespoke and standardised information systems for liaison psychiatry services, the development of which could be led by the Royal Colleges of Psychiatry, Nursing, Medicine and Emergency Medicine. A standardised system of recording for liaison psychiatry would also enable easier benchmarking across services for quality and performance management.

There has been a national commitment by government in England to work towards greater integration of health and social care, with a particular focus on placing the individual at the centre of change and the person around whom, services should be planned [26]. The government departments of health and social care have also merged recently in order to pursue greater joined up care. Although there are many different definitions of integrated care, most encompass the principles discussed in this paper, including a patient focus, smooth inter and intra-professional team working and a shared vision of care.

It is interesting that in the interviews with liaison and acute hospital staff, performance monitoring did not emerge as a theme, although it is considered an important component of delivering effective integrated services (). It’s absence from any of the discourse during the interviews we carried out suggests it may require a higher profile and greater buy-in from staff.

**Fig. 1:**
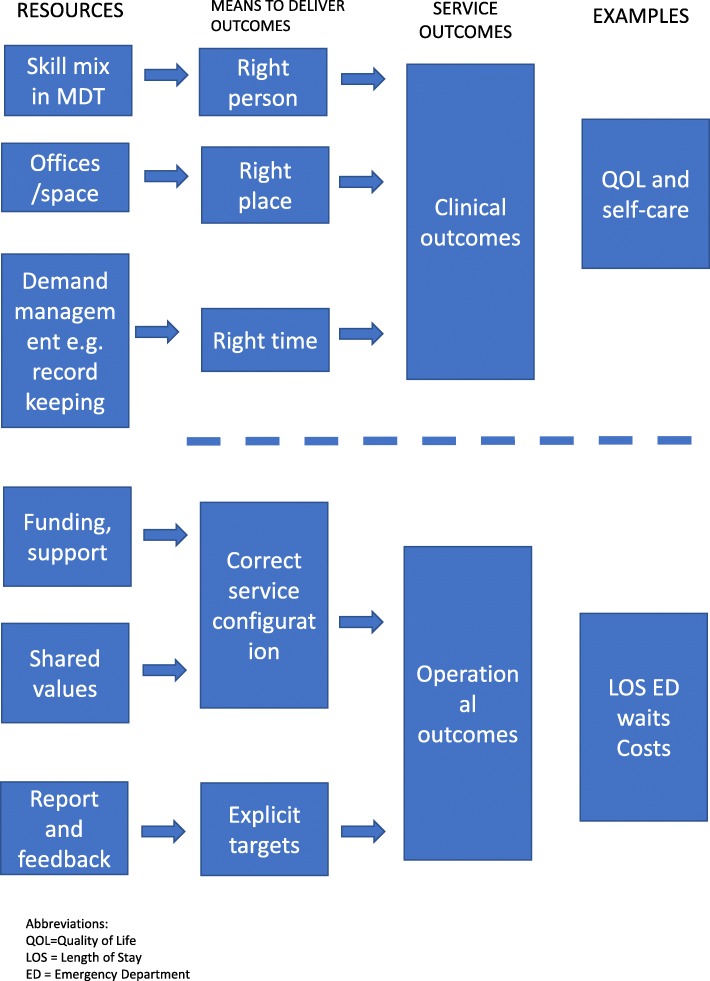
Diagrammatic representation of the resources required, means to deliver outcome, and service outcomes for integrated liaison services

Our findings can be summarised in diagrammatic form as shown in Fig. 1. Integrated liaison services require key resources, plus an organisational structure to deliver timely assessment and treatment, in order to deliver key outcomes (clinical or system focused). Services need to be located on site in the acute hospital, have sufficient staff and skill-mix to meet demand, and have shared patient-focused goals within the team and with acute hospital staff and community staff they work with. They need to be able to record information and monitor referral and outcome in an efficient manner which is tailored to the high volume/low intensity nature of their work.

### Strengths and limitations

This study consisted of a relatively large sample of liaison practitioners from services which were representative of the four most common types of service in England at present. We deliberately sampled from the four different service types, to ensure we obtained the views of staff working in services of different sizes, working patterns, acuity, serving different age populations, coverage of the acute hospital and acute versus less acute care. The interviews consisted of open questions about services, but the themes that emerged predominantly concerned factors related to integration of teams within the hospitals in which they worked. The advantage of organising the themes according to the domains described by Suter and colleagues is that all these domains have to a greater or lesser degree tools that can be used to measure performance [10]. Thus, many of the concerns raised by staff in qualitative interviews can be objectively measured in future work.

Out of the 168 liaison services we initially approached, only 13 actually sent representatives to the initiation workshops we set up. It is possible therefore that the services from which we sampled were different in some way to those who declined to participate or did not respond to our invitation. It is possible that teams who agreed to participate in the current study are high functioning services who are open to scrutiny, in which case our findings may under-estimate problems and difficulties in liaison services. Equally, it can be argued that services facing a high degree of problems and difficulties wished to participate in order to secure a platform for their discontent, in which case our findings may over-estimate problems in liaison services in England.

We interviewed a relatively smaller number of acute hospital staff than liaison staff, and the hospital staff that were interviewed were sourced by the liaison teams. It is possible therefore that the teams identified hospital staff with a favourable attitude to liaison services, and again our findings may underestimate problems and difficulties. Despite this, the hospital staff we interviewed, were able to raise concerns, and spoke openly and freely about problem areas and difficulties with the liaison team, with which they interacted.

Another limitation of this study, is that we did not interview any service users about their views of liaison services. This is something we are planning to do in a separate phase of LP-MAESTRO.

## Conclusion

Liaison mental health staff working in liaison mental health services in England and acute hospital staff, working in hospitals served by liaison psychiatry teams, have identified a series of barriers to providing integrated mental and physical health care, in several key domains including: problems with continuity of care across the secondary-primary interface; a lack of mental health resources in primary care to support discharge; a lack of shared information systems and a disproportionate length of time spent recording information as opposed to face to face patient contact; and a lack of a shared vision of care. There was evidence of good inter-professional team working, placing the patient at the heart of the process, and support for the development and expansion of liaison services by commissioners and other relevant stakeholders.

## Additional file

Additional file 1: Topic Guide. Topic Guide used for clinicians in the LP-Maestro study. (DOCX 58 kb)

## Data Availability

Data is not available due to the requirement to protect the confidentiality of the participants.

## Abbreviations

CMHT: Community Mental Health Teams
COREQ: Consolidated criteria for reporting qualitative research
CPMS: Central Portfolio Management System
ED: Emergency Department
IAPT: Improving Access to Psychological Treatment
LP: Liaison Psychiatry
MUS: Medically unexplained symptoms
RAID: Rapid Assessment Interface Discharge
