# Supplementary material for: Integrated liaison psychiatry services in England: a qualitative study of the views of liaison practitioners and acute hospital staffs from four distinctly different kinds of liaison service
Source: BMC Health Serv Res. 2019 Jul 25;19:522. doi: 10.1186/s12913-019-4356-y (PMC6659268; doi:10.1186/s12913-019-4356-y)
Supplement: Supplementary file 1 — Topic Guide. Topic Guide used for clinicians in the LP-Maestro study. (DOCX 58 kb) [file 12913_2019_4356_MOESM1_ESM.docx]

**LP-MAESTRO: Topic guide CLINICIANS**

**Aims and objectives**

- **History**: Provide an in-depth account of how diverse configurations of liaison psychiatry came to exist.
- **Delivery**: Develop a detailed understanding of what LP services do in different settings now.
- **Desired outcomes**: Map what outcomes matter in different settings/service components and how they are achieved (ED, wards, OP clinics).
- **Future**: Explore what services think they should do and look like in the future.

**Use of topic guide**

The topic guide includes prompts and probes for interviewers rather than fully formed questions. The guide is designed to be used flexibly to allow interviewers to be responsive to issues raised by participants. Responses to questions will be probed fully, with researchers asking ‘why’ throughout.

**Section 1: Introduction** (3 minutes)

*Aim: Explain the research, ensure informed consent and answer any questions.*

- Introduce self
- Purpose – brief explanation of LP-MAESTRO and purpose of this interview:
  - **History and influence:** Historical and current influences on what the service is and how it operates
  - **Delivery:** What the service is supposed to do, and what it actually does – people seen, types of response, expected outcomes.
  - **Future:** Thoughts looking ahead – ambitions, plans, expectations
- Explain voluntary nature
- Confidentiality and anonymity – everything is confidential, might use quotations in written reports not possible to identify individuals
- Recording – would like to audio record with permission, data stored in accordance with the Data Protection Act
- Timings – reiterate start and finish times
- Any questions?

**START RECORDING – record verbal consent**

**Section 2: Background and context** (10 minutes)

*Aim: Get participants talking and establish helpful contextual information.*

**Background**

**Professional background**

- Briefly explore professional background (what do they do, how long for, length of time working in current hospital/setting).

**Role in/with LP**

- Explore their role in the LP team/how they work with them, probe on:
  - - What they do day-to-day (how far days vary/are the same)
    - Length of time in that role/working with LP team

**Personal history**

- Map how participant came to work for LP service, *probe on:*
  - - How/when they first heard about LP
    - What previous experiences of LP involved
    - How ended up in current role (*active decision, fell into?)*

**Context – make-up of the LP service**

*Researcher note: We do not need to cover low priority areas with all participants. When we have started to build a good picture of the service please skip.*

- (low priority) Map different settings the LP service works in (*ED, wards, outpatient clinics, specialist services, other?)*

- (low priority) Identify characteristics of the service, *probe on:*
  - whether there are separate teams for older/working age adults, how/who provides any out of hours services
- Explore relationship the LP team has with overlapping services, *probe on:*

- - any links with drug and alcohol services, or psychology
  - whether/how LP team refers to other services during stay in hospital
- Map any non-clinical services provided (education/training/other non-clinical activities)

**Section 3: Delivery** (20 minutes)

*Aim: To develop a detailed picture of the types of clinical work done in different settings and how LP differs to other services (SEE ATTACHED SCENARIO SHEET)*

***Researcher note:*** Ask participants to provide illustrative examples throughout this section.

*Make clear that we do not want them to compromise patient confidentiality.*

**Different clinical settings**

Probe on experiences of the following clinical problems:

- Repeat presentations in ED after self-harm
- Dementia which does not explain current admission
- Poor self-management of chronic conditions (e.g. diabetes)

*Explore each relevant clinical setting in turn.*

**Out-patient clinics Emergency Department**

**Non-urgent wards Specialist services**

***Context***

- Explore what the LP team does in this setting, *probe on:*
  - Requests and referrals
    - What is the process
    - Who refers/what sort of working relationship do they have
  - Types of patient – older age/working age
  - Types of clinical problem the LP service sees

***Actions***

- What do they do/how they respond to different types of problem, *probe on:*
  - Processes/treatments available (psychological therapies, transfer, rapid response, other)
  - How far responses vary (e.g. staff availability/other factors)
  - Who delivers different treatments, who carries out assessments
  - When are services available/what cover at weekends/evenings
  - Whether refer onto other services
  - Any duplication of services (e.g. same cases ever referred to clinical/health psychology)

***Outcomes***

- Explore what happens as a result of their actions, *probe on:*
  - For patients
    - Re-admittance less likely, improved health, other?
  - Clinicians who refer
    - Reduced LOS, financial savings, other?
- Explore what ‘ideal’ outcomes look like in this setting
- How far are these achieved
- What supports/helps them being achieved
- What challenges/constraints limit achieving successful outcomes *probe on:*
  - - Resources
    - Working relationships (tensions)
    - Systems/processes
    - Other constraints?
- Explore any examples of overcoming challenge/constraints

**Section 4: Service history and ongoing influences** (15 minutes)

*Aim: To explore ongoing influences that have shaped, and continue to shape the service.*

*Researcher note: some of the below will be more/less relevant depending on participant type - important to start broad. Be mindful of potential limited knowledge around service history.*

**Service history**

- Explore history of the service, *probe on:*
  - - When the LP service/ different components were set-up
    - Who set-up/was involved in set-up
    - Why was the service/different components set-up, *probe on:*
      - A specific incident
      - Strategic/proactive decision
      - Organic/ad-hoc nature
      - Other?

**Ongoing influences**

- Map what they think are the key influences on the service/how it functions, *probe on:*

*Researcher note:* Fully probe each factor identified.

- - CCG/Commissioners (any expectations/KPIs)
  - Acute Trust
  - Mental Health Trust
  - Providers of other related or overlapping services, including social services
  - Factors like local location of units;
  - Important local individuals; local specific eg untoward incidents
  - Characteristics of team members – experience, skills, attitudes.
  - The national scene – NHS England, RCPsych
  - Resources/funding (*probe on any variations in funding/commissioning for different service components)*
  - Other factors

**Section 5: Future of Liaison Psychiatry** (10 minutes)

*Aim: To explore views of what LP should look like in the future (local and nationally).*

**Local**

- Explore how they envisage their service in the future, *probe on:*
  - Whether will stay same/change
  - Why?
  - Response (what do they think about this)?

**National**

- What do they think LP should look like in the future, *probe on*
  - Staff make-up/professional backgrounds
  - Hours/days of coverage
  - Acute pathway care only
  - RAID
  - Outpatient clinics
  - Therapy
  - CORE, CORE24, ENHANCED, COMPREHENSIVE *(explore usefulness of these terms)*

**Section 6: Conclusion and wind-down** (3 minutes)

*Aim: To give participants a chance to add any final thoughts and wind down before the end of the interview.*

- Explore the single most important thing participants think liaison psychiatry does.
- What one thing should any future LP research do (what should it measure?).
- Check whether anything to add.

**END RECORDING**

- Wind down
- Reassure about confidentiality
- Check whether any questions
